# Digoxin toxicity with therapeutic serum digoxin concentrations

**DOI:** 10.1016/j.toxrep.2025.102079

**Published:** 2025-07-05

**Authors:** J. Graafsma, N. Cimic, M. Dijkman, F.M.J. Gresnigt, D. Mitrovic, C. Smit

**Affiliations:** a Department of Hospital Pharmacy, Frisius MC, Heerenveen, the Netherlands; b Department of Clinical Pharmacy and Pharmacology, University Medical Center Groningen, Groningen, the Netherlands; c Department of Intensive Care Medicine, Frisius MC, Heerenveen, the Netherlands; d Dutch Poisons Information Center, UMC Utrecht, Utrecht, the Netherlands; e Department of Emergency Medicine, OLVG, Amsterdam, the Netherlands; f Department of Clinical Pharmacy, Antonius hospital, Sneek, the Netherlands

**Keywords:** Digoxin toxicity, Digoxin antibodies, Renal failure, Hyperkalemia

## Abstract

**Introduction:**

Digoxin is a cardiac glycoside used for rate control in atrial fibrillation and heart failure. Despite its efficacy, digoxin has a narrow therapeutic window and can cause severe side effects, including life-threatening arrhythmias. Literature and guidelines on management of digoxin toxicity remain inconsistent whether to include serum digoxin concentrations as a key criterium for diagnosing digoxin toxicity and determining the indication for digoxin-specific antibody fragments. This report presents a case of digoxin toxicity at therapeutic serum concentrations.

**Case report:**

A 76-year-old male presented with bradycardia, hyperkalemia, and acute kidney injury following gastrointestinal bleeding. Despite serum digoxin concentrations within the therapeutic range (1.4 ng/ml), the patient exhibited symptoms consistent with severe digoxin toxicity. Initial treatments, including calcium gluconate, insulin-glucose, and sodium bicarbonate, failed to resolve hyperkalemia and/or bradycardia. Administration of 40 mg digoxin-specific antibody fragments led to rapid normalization of potassium levels, improved heart rate, and hemodynamic stabilization, indicative for severe digoxin toxicity despite therapeutic serum concentrations.

**Discussion:**

This case demonstrates that digoxin toxicity can occur at serum concentrations in therapeutic range, emphasizing the importance of clinical features in diagnosing digoxin toxicity. Current guidelines vary on the role of serum digoxin concentrations in guiding the use of digoxin-specific antibody fragments, but this case underscores its efficacy in resolving symptoms related to digoxin toxicity, even at low serum concentrations.

## Highlights

• Digoxin toxicity can occur at therapeutic serum concentrations.
• Clinical symptoms are crucial in diagnosing digoxin toxicity next to serum levels.
• Digoxin-specific antibody fragments resolved severe toxicity at low serum concentrations.
• Current guidelines vary on the role of serum digoxin concentrations in toxicity management.

## Graphical Abstract

**Figure:**
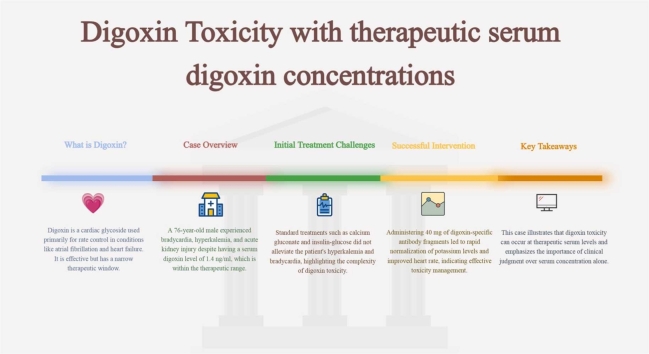

## 1 Introduction

Digoxin is a cardiac glycoside that exerts several key effects on the heart. It enhances myocardial contractility (positive inotropic effect), inhibits atrioventricular (AV) conduction (negative dromotropic effect), and reduces heart rate (negative chronotropic effect). These effects are achieved through inhibition of the sodium-potassium pump (Na-K-ATPase), which leads to an intracellular increase in sodium ion concentrations and a decrease in potassium ion concentrations. Additionally, calcium influx in the cardiomyocytes increases, which enhances the force and velocity of myocardial contractions, as well as cardiac output.

Digoxin is an old drug, used as a rate controlling drug in atrial fibrillation and heart failure. Despite a small therapeutic window, reports of severe side effects and limited evidence on its therapeutic value, digoxin is still recommended in European heart failure and atrial fibrillation guidelines, predominantly when rate control is desired [1], [2]. In the Netherlands, about 75,000 people are currently using digoxin [3]. Clinical use of digoxin can be complicated by severe side effects such as life-threatening arrhythmias, which can be effectively treated using digoxin-specific antibody fragments [4]. Raised serum digoxin concentrations above 2 ng/ml, in the presence of specific clinical features were found to be strongly suggestive of digoxin toxicity [5], although this relationship has been challenged in later studies [6], [7]. National guidelines and expert recommendations on management of digoxin toxicity remain inconsistent regarding the inclusion of serum digoxin concentrations as a key criterion for diagnosing digoxin toxicity and determining the indication for digoxin-specific antibody fragments [8], [9], [10]. Especially, the value of serum concentrations below the toxic threshold in moderate to life-threatening toxicity is subject of debate. Here, we present a case illustrating the potential role of digoxin-specific antibody fragments for potential life-threatening digoxin toxicity with low serum digoxin concentrations.

## 2 Case

**Fig. 1:**
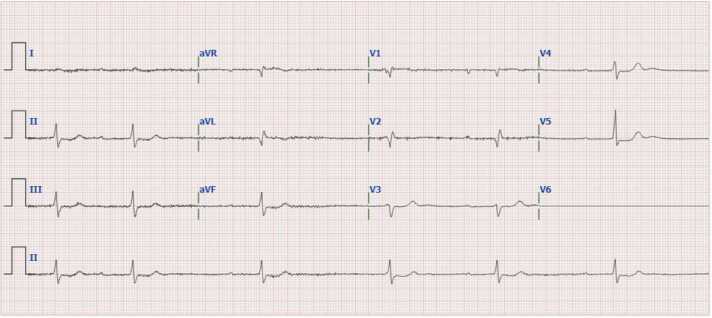

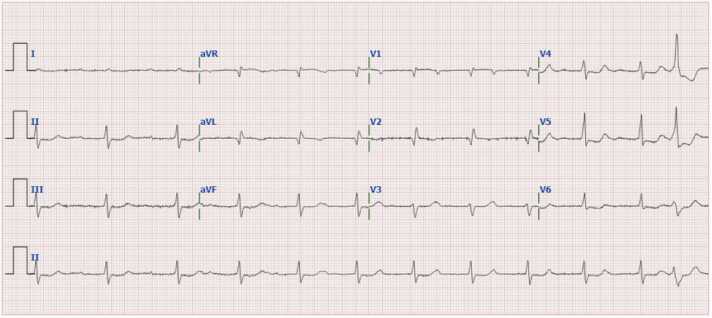
(**a**) ECG obtained four minutes before administration of DigiFab® showing a sinus rhythm with frequent episodes of sinus arrest. A variable complete atrioventricular (AV) block is present, with periods of an AV junctional escape rhythm at a rate of 35 beats per minute, alternating with episodes of high-grade AV block, indicating markedly impaired atrioventricular conduction. The QRS complexes demonstrate an intermediate frontal axis and have a duration of 110 ms. Morphologically, a qR complex is observed in leads V1, V2, and aVL. R-wave progression across the precordial leads is poor, and no discernible signal is present in lead V6. Low QRS voltages are noted, with amplitudes less than 5 millimeters in the limb leads and up to 10 millimeters in the precordial leads. Regarding repolarization, there is a 1-millimeter ST-segment depression in the lateral leads. The P waves are of low amplitude but not notched. The QT-interval is within normal limits. Fig. 1**b** ECG obtained about 7.5 h after administration of DigiFab® showing a sinus rhythm at a rate of 68 beats per minute. There is marked first-degree atrioventricular (AV) block, with a prolonged PR interval of 300 ms. One-to-one AV conduction is preserved. The frontal axis, QRS voltages, depolarization abnormalities, and repolarization abnormalities remain unchanged compared to the previous ECG. Additionally, a single ventricular extrasystole is observed.

**Fig. 2:**
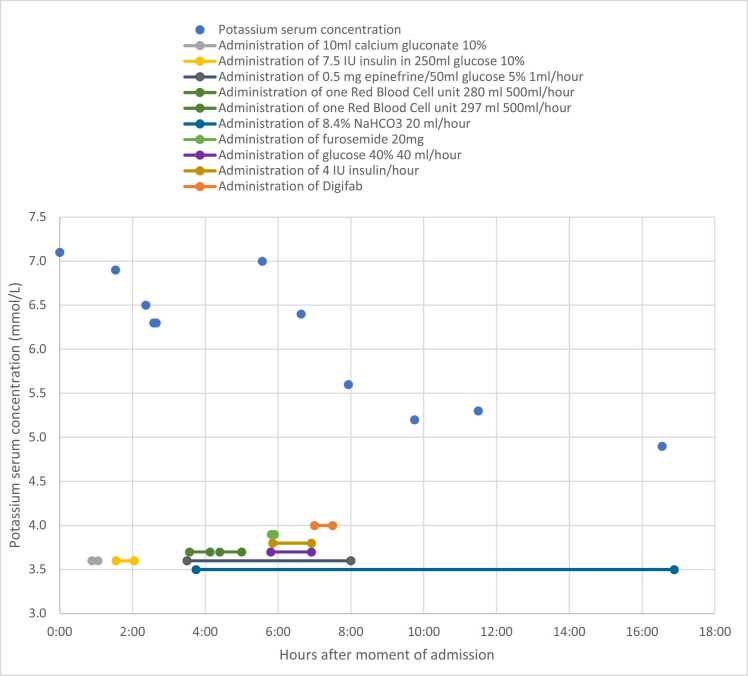
Serum potassium concentration and administered comedication/infusion therapies in the first 18 h after admission.

A 76-year old male (86 kg) was admitted to the emergency department with melena and a decreased hemoglobin level, severe bradycardia, hyperkalemia and an acute on chronic kidney insufficiency. The medical history included heart failure, atrial fibrillation, a MitraClip procedure and wild-type transthyretin amyloidosis (wtATTR-CM). Prescription medication included allopurinol, bisoprolol, furosemide, lisinopril, rivaroxaban, tafamidis, triamcinolone cream and digoxin in a dosage of 0.0625 mg once a day. On presentation to the emergency department, the patient reported feeling short of breath and fatigued, but otherwise did not feel ill or nauseous. At admission the patient appeared somewhat pale with hypotension (99/65 mm/Hg) and bradycardia (39 beats per minute). During the first hours of admission, systolic and diastolic blood pressure varied between 91 and 112 mmHg and 45–55 mmHg, respectively. Throughout this period, heart rate remained around 50 beats per minute, although some moments the heart rate was down to 35 beats per minute. Electrocardiogram (ECG) showed no P-wave abnormalities, a normal axis, no ST-deviation, a QRS-duration of 140 ms, a corrected QT interval of 442 ms and a variable complete atrioventricular (AV) block with episodes of high-grade AV block (Fig. 1a). No previous ECG’s were available to compare these findings. Laboratory measurements (Fig. 2, Table 1) showed a hyperkalemia (7.1 mmol/L), renal dysfunction (estimated GFR 11 ml/min/1.73 m^2^, which was 44 ml/min/1.73m^2^ five months before admission) and a reduced hemoglobin level (5.9 mmol/L). Other physical examination and laboratory results were unremarkable.

**Table 1:** Relevant laboratory investigations the first 37 h after admission (local reference values shown between brackets).

**Lab values (reference)→** **(hh:mm) ↓**	eGFR*^1^ **(>60 ml/ min/ 1.73 m2)**	**Creatinine**^**1**^ **(50–110 µmol/L)**	**Hemoglobin**^**1**^ **(8.5–11.0 mmol/L)**	**Sodium**^**1**^ **(136–145 mmol/L)**	**Urea**^**1**^ **(2.5–7.5 mmol/L)**	**Bicarbonate**^**1**^ **(22–29 mmol/L)**	**Lactate**^**1**^ **(<2 mmol/L)**	**pH**^**2**^ **(7.35–7.45)**	**pCO2**^**2**^ **(4.7–6.4 kPa)**	**pO2**^**2**^ **(10.0–13.3 kPa)**
**00:05**	11	423	5.9	140	63.7					
**02:22**	13	376	5.5	135	61.6					
**02:35**						9	1.0	7.28	2.6	16.1
**05:34**						10	1.0	7.30	2.7	15.4
**06:38**						11	1.4	7.34	2.8	14.0
**07:56**						12	1.1	7.38	2.6	15.0
**09:45**						14	0.9	7.38	3.2	13.8
**11:30**	15	324			53.2					
**12:00**						15	1.0	7.43	3.0	15.6
**16:33**						18	1.1	7.48	3.1	12.7
**24:30**	19	263	6.2	140	42.9					
**37:00**	19	263	6.5	143	38.4					

*The eGFR was calculated using the CKD-EPI equation ^1^ Determined in serum ^2^ Determined in blood gas

The patient was diagnosed with a gastro-intestinal bleeding, which was probably existing for a longer time already, most likely provoked by the use of rivaroxaban in combination with a deteriorated renal function, which was treated with prothrombin complex concentrates (Octaplex®) in line with local protocols. No melaena was observed after the administration of Octaplex®. Hyperkalemia was immediately treated with 10 ml of calcium gluconate 10 % and 7.5 International Units (IU) of insulin in 250 ml of 10 % glucose. This treatment resulted in a small decrease in serum potassium to 6.3 mmol/L (Fig. 2). About two hours after admission the patient was transferred to the intensive care unit (ICU), primarily due to persisting high potassium levels and bradycardia. There, epinephrine and bicarbonate infusion were initiated (Fig. 2). Additionally, glucose (40 % 40 ml per hour) and insulin (four IU per hour) were continuously administered by infusion. Furthermore, the patient received two units of red blood cell (RBC) concentrate and normal saline, a total of 1200 ml of fluids. During and after the administration of these treatments the patient remained hemodynamically instable, bradycardia worsened to 35 beats per minute, and hyperkalemia persisted (>6.3 mmol/L) despite continuous epinephrine infusion and the administration of bicarbonate and insulin. With admission to the ICU, digoxin toxicity was considered and logistics measures were commenced to obtain digoxin-specific antibody fragments, which is centrally stored in a national calamity stock in the Netherlands,.

The serum concentration of digoxin was 1.2 ng/ml (therapeutic reference values 0.8 – 2.0 mg/L), in a blood sample drawn 2.5 h after admission. However, the combination of a deteriorated renal function and worsening bradycardia and hyperkalemia despite the administration of insulin, glucose and calcium gluconate, strengthened digoxin toxicity as working diagnosis. Then one vial of Digifab® containing 40 mg digoxin-specific antibody fragments was administered about 6.5 h after admission. The administration of insulin and glucose were discontinued, and administration of calcium gluconate and furosemide were already discontinued. Within 30 min after the administration of digoxin-specific antibody fragments, and without any other additionally therapy (except for fluids and continuous epinephrine infusion), the heart rate increased from 35 to 50 beats per minute and the epinephrine infusion could be stopped as the patient stabilized hemodynamically. Furthermore, a rapid substantial decrease in serum potassium was witnessed, which eventually returned to normal values (Fig. 2).

The ECG after the administration of digoxin-specific antibody fragments still shows a first degree AV block (Fig. 1b), but improved atrioventricular conduction compared to the first ECG (Fig. 1a).

About 24 h after admission the patient was transferred to a general ward and discharged after six days. Since spontaneous rate control was achieved upon discontinuation of digoxin, treatment with digoxin was permanently stopped.

## 3 Discussion

This case report demonstrates that chronic digoxin toxicity may occur while serum digoxin concentrations are within the therapeutic range. Administration of digoxin-specific antibody fragments had an immediate and clinically relevant effect on a life-threatening hyperkalemia and bradycardia in this 76-year-old patient, therefore a serious digoxin toxicity was suspected. It should be acknowledged that, besides digoxin toxicity, other factors such as beta-blockade usage might have played an additional role in the clinical presentation of this case. The digoxin toxicity occurred while the serum digoxin concentration was within therapeutic range and this case shows that in these situations, administration of digoxin-specific antibody fragments can be of great clinical importance.

During treatment, a serum digoxin concentration of 1.2 ng/ml was measured. To rule out an erroneous measurement, a second digoxin serum concentration was determined retrospectively in a blood sample taken 2,5 h earlier, upon admission to the emergency department. At that time, no therapy had been initiated yet. This measurement showed a concentration of 1.4 ng/ml, which is also within therapeutic limits. The difference of 0.2 ng/ml between the two samples could be explained by a simple assay variability or dilution due to excessive infusion therapy during the first hours of admission. The elimination half-life of digoxin would be extended up to longer than 100 h due to the deteriorating renal function, and would not explain this decrease [13].

Digoxin toxicity is generally associated with serum concentrations of ≥ 2.0 ng/ml and can be severe with serum concentrations of ≥ 4–5 ng/ml [11], [12], [13]. Nevertheless, studies demonstrated digoxin toxicity with serum concentrations of 1.5–2.0 ng/ml [12]. To the best of our knowledge, this is the first case report describing severe life-threatening digoxin toxicity with serum concentrations below 1.5 ng/ml, treated successfully with digoxin-specific antibody fragments.

The patient described in this case report showed some well-known risk factors for digoxin toxicity, like older age and an acute on chronic renal insufficiency [9]. An interesting additional note is that the patient was known with cardiac amyloidosis. Amyloidosis is characterized by extracellular protein fibril deposition throughout the body’s organ systems. Atrial and ventricular arrhythmias and conduction diseases are common in cardiac amyloidosis [14], [15]. However, digoxin as a pharmacological therapy for rate control is considered to be poorly tolerated and should be used with caution in cardiac amyloidosis. Rubinow et al. showed that digoxin binds avidly to amyloid fibrils in vitro. This binding might lead to increased tissue and serum drug levels, prolonged exposure of digoxin receptors and a high risk of digoxin toxicity [14], [15], [16], [17]. This indicates that this patient potentially exhibited a higher risk for digoxin toxicity.

Visual disturbances, nausea, diarrhea, confusion and lethargy are symptoms that can be noticed in digoxin toxicity, but were not seen in this patient [9]. However, this patient exhibited various symptoms indicative for life-threatening digoxin toxicity, namely hyperkalemia, bradycardia and hypotension. To be able to resolve these symptoms, several treatments were commenced; administration of epinephrine, sodium bicarbonate, insulin in glucose and calcium gluconate. Calcium counteracts the increase of the threshold potential of the cardiac myocytes due to the hyperkalemia, and thereby protects the heart against fatal dysrhythmias. The administration of calcium gluconate in digoxin toxicity has been challenged in literature, as it could worsen dysrhythmias, but more recent studies show that the most important determinants in calcium gluconate administration are the dose and rate of infusion, regardless the presence of digoxin toxicity [18], [19]. Insulin is known to decrease the serum potassium level by 0.6–1.2 mmol/L within minutes [20]. Initially, administration of insulin and glucose resulted in a decrease in serum potassium level, although hyperkalemia persisted and even increased over time despite continuous infusion of insulin and glucose. So, an initial effect of insulin was witnessed, but did not persist, indicating that the effect of the high dose continuous insulin therapy was insufficient in this case, and therefore discontinued. Due to a persisting hyperkalemia combined with the continuous severe bradycardia, despite continuous epinephrine infusion, digoxin toxicity was considered and digoxin-specific antibody fragments were administered, despite a normal digoxin serum concentration. Before the administration of the digoxin-specific antibody fragments, all other initiated treatments were discontinued (insulin, glucose, calium gluconate), except for fluids and continuous epinephrine infusion. The administration of the digoxin-specific antibody fragments resulted in a rapid decrease in serum potassium and improvement of the bradycardia and hemodynamics, suggesting that these effects were due to the digoxin-specific antibody fragments. Due to the improvement of the bradycardia and the blood pressure, the continuous epinephrine infusion could also be stopped, which was not possible earlier.

Other clinical features, such as bisoprolol toxicity or BRASH syndrome, cannot be ruled out completely. The patient experienced bradycardia and hypotension, which are symptoms consistent with bisoprolol toxicity. However, we expect that bisoprolol toxicity played a minor role in this case, as a normal maintenance dose was used (10 mg once a day), and approximately 50 % of bisoprolol it metabolized through the liver into inactive metabolites, which implies that the impact of an acute reduced kidney function on the excretion of bisoprolol is not clinically significant. Bisoprolol might have played a synergistic role in the bradycardia and hypotension, but bisoprolol toxicity as a sole cause is considered unlikely. Patients with BRASH syndrome exhibit the following symptoms: bradycardia, renal failure, AV nodal blockade, shock and hyperkalemia, which are all symptoms consistent with this case. Treatments for both bisoprolol toxicity as BRASH syndrome include calcium gluconate, insulin and epinephrine, which were all administered without effect. However, as described above, the bradycardia and hyperkalemia did resolve rapidly after the administration of digoxin specific antibodies even though the other therapies were already discontinued. This underscores the prominent role of digoxin toxicity in this case.

Several studies report that the serum concentration of digoxin is not the sole criterium in diagnosing digoxin toxicity, as toxicity and serum concentrations are not always correlated [21]. For example, a retrospective study by Park et al. showed that out of 123 patients with a serum digoxin concentration greater than 3 ng/ml, 54 had no apparent signs or symptoms of toxicity at the time the serum digoxin concentration was determined [22]. Other studies underscore the conclusion that high serum digoxin concentrations do not provide a definitive diagnosis of digoxin toxicity [12], [23]. Additionally, when symptoms of digoxin toxicity are clinically present, serum digoxin concentrations may be within therapeutic range. A prospective study by Ong et al. in 67 patients, showed that patients with clinical features of digoxin toxicity, such as cardiac arrhythmias, had a significantly higher serum digoxin concentration compared to the non-toxic group (2.09 ± 1.28 ng/ml vs. 1.20 ± 0.75 ng/ml). However, there was a considerable overlap of serum digoxin concentrations between these two groups, indicating that clinical features of digoxin toxicity can be present without elevated digoxin concentrations [24].

Current guidelines on management of digoxin toxicity vary in their stance on whether serum digoxin concentrations should be considered a key factor in diagnosing digoxin toxicity and deciding the need for digoxin-specific antibody fragments [8], [9], [10]. Especially, the added value of digoxin-specific antibody fragments is unclear when digoxin serum concentrations are below the usual toxic threshold. The fact that guidelines are inconsistent on this matter is in line with previous studies that demonstrate the lack of a clear relationship between serum concentrations and digoxin toxicity. This case report underscores the importance for diagnosing digoxin toxicity based on clinical features, without ascribing too much value to digoxin serum concentrations when they are below the toxicity threshold. In our opinion, the decision to administer digoxin-specific antibody fragments should primarily be based on clinical features, with only a minor role for digoxin serum concentrations.

This case report shows that the role of serum digoxin concentrations in digoxin toxicity is limited. We recommend that in case of potentially life-threatening digoxin toxicity, serum digoxin concentrations should be interpreted with caution in clinical decision-making regarding diagnosis and antidote administration.

## Informed consent

Informed consent was obtained from the patient. Possible identifying information was excluded in this article.

## Funding

This research did not receive any specific grant from funding agencies in the public, commercial, or not-for-profit sectors.

## Declaration of Competing Interest

The authors declare that they have no known competing financial interests or personal relationships that could have appeared to influence the work reported in this paper.

## Data Availability

No data was used for the research described in the article.
